# The Elimination of the Inebriate

**Published:** 1903-11-07

**Authors:** 


### THE ELIMINATION OF THE INEBRIATE.

INEBRIETY is a disorder of great antiquity and it seems likely long to remain as evidence of the frailty of human nature. It is certainly a remarkable fact that man in almost all stages of his upward climb has manifested a disinclination to limit his drink to water. In the course of his evolution he has sought out many inventions; some have proved beneficial, but not a few have been highly detrimental. Very early man must have found out the curious influence exerted by fermented liquors, and, once discovered, the attraction which lurks about alcoholic beverages has not been allowed passage to the things forgotten. And so we find among most people, with the exception of some few still in the pre-agricultural stage, like the aborigines of Australia, inebriety acting as a deranging influence in social affairs, and working much physical, mental, and moral deterioration in the individual. It is unnecessary to discuss the contention that the introduction of various forms of alcoholic beverages has on the whole proved beneficial in the evolution of the human. Whether this be so or not, it is clear that in the abuse of alcohol man has had to contend with one of the most powerful influences making for racial deterioration and arresting national progress.

Fortunately of recent years the subject has received careful scientific study, and at the present time much attention is being devoted to a serious investigation of the pathology of the condition. For some time efforts, often ill-advised and unreasonable in their manner of application, have been carried on to secure satisfactory cure of the inebriate. Unfortunately the condition is one which has many attractions for the quack and unscrupulous speculator, and the inebriate has been exploited by many frauds promising restoration by "secret" cures. Institutions of all kinds established with a view to afford the drunkard a re-establishment or re-creation of will power have come into being, but as our Special Medical Commissioner is showing in the series of articles, now attracting much attention, on British Institutions for the Care of the Inebriate, these establishments are by no means always ideal institutions, but like mankind may be classed as good, bad, and indifferent.

The fact is that much in our methods of dealing with the inebriate is purely empirical, and many of our measures are in great part experimental. The pathogeny of the condition needs elucidating, and much further light is required on the pathology of the morbid state before we can claim for our efforts at restoration anything like a rational and scientific precision. Until all the ætiological factors influential in the initiation, establishment, and maintenance of inebriety are more clearly revealed prophylaxis must in great measure proceed haltingly; and until we break away from mediæval conceptions regarding its essential nature treatment is likely to remain lacking in the most desirable elements. Many suggestions have been made with a view to secure the speedy elimination of the inebriate, but most of these are either such as cannot be enforced without resigning present day views regarding personal liberty or departing from modern standards of humanitarian action, and even on scientific grounds serious objections may be presented. Modern conceptions regarding the nature of heredity have also gone far to modify our views regarding the likely fate of the inebriate's offspring; and it must be admitted that drunkards, not a few paroxysmal, have proved themselves among the most valuable assets of the State.

At the present time it is necessary to keep an open mind regarding action relating to the care or custody of the victim of alcohol. It is to be hoped that such scientifically directed bodies as the British and American Societies for the Study of Inebriety, and the various committees in connection with county and municipal councils formed to consider the action of the Inebriates Acts, will lose no time in collecting all data which may have directing value in indicating the best way to eliminate inebriety.

